# Neonatal Brain Tissue Classification with Morphological Adaptation and Unified Segmentation

**DOI:** 10.3389/fninf.2016.00012

**Published:** 2016-03-29

**Authors:** Richard J. Beare, Jian Chen, Claire E. Kelly, Dimitrios Alexopoulos, Christopher D. Smyser, Cynthia E. Rogers, Wai Y. Loh, Lillian G. Matthews, Jeanie L. Y. Cheong, Alicia J. Spittle, Peter J. Anderson, Lex W. Doyle, Terrie E. Inder, Marc L. Seal, Deanne K. Thompson

**Affiliations:** ^1^Murdoch Childrens Research Institute, The Royal Children's HospitalMelbourne, VIC, Australia; ^2^Department of Medicine, Monash Medical Centre, Monash UniversityMelbourne, VIC, Australia; ^3^Department of Neurology, Washington University School of MedicineSt. Louis, MO, USA; ^4^Department of Psychiatry, Washington University School of MedicineSt. Louis, MO, USA; ^5^Florey Institute of Neuroscience and Mental HealthMelbourne, VIC, Australia; ^6^Department of Paediatrics, University of MelbourneMelbourne, VIC, Australia; ^7^Royal Women's HospitalMelbourne, VIC, Australia; ^8^Department of Obstetrics and Gynaecology, University of MelbourneMelbourne, VIC, Australia; ^9^Department of Physiotherapy, University of MelbourneMelbourne, VIC, Australia; ^10^Department of Pediatric Newborn Medicine, Harvard Medical School, Brigham and Women's HospitalBoston, MA, USA

**Keywords:** magnetic resonance imaging, tissue classification, statistical parametric mapping, neonate, preterm birth

## Abstract

Measuring the distribution of brain tissue types (tissue classification) in neonates is necessary for studying typical and atypical brain development, such as that associated with preterm birth, and may provide biomarkers for neurodevelopmental outcomes. Compared with magnetic resonance images of adults, neonatal images present specific challenges that require the development of specialized, population-specific methods. This paper introduces MANTiS (Morphologically Adaptive Neonatal Tissue Segmentation), which extends the unified segmentation approach to tissue classification implemented in Statistical Parametric Mapping (SPM) software to neonates. MANTiS utilizes a combination of unified segmentation, template adaptation via morphological segmentation tools and topological filtering, to segment the neonatal brain into eight tissue classes: cortical gray matter, white matter, deep nuclear gray matter, cerebellum, brainstem, cerebrospinal fluid (CSF), hippocampus and amygdala. We evaluated the performance of MANTiS using two independent datasets. The first dataset, provided by the NeoBrainS12 challenge, consisted of coronal *T*_2_-weighted images of preterm infants (born ≤30 weeks' gestation) acquired at 30 weeks' corrected gestational age (*n* = 5), coronal *T*_2_-weighted images of preterm infants acquired at 40 weeks' corrected gestational age (*n* = 5) and axial *T*_2_-weighted images of preterm infants acquired at 40 weeks' corrected gestational age (*n* = 5). The second dataset, provided by the Washington University NeuroDevelopmental Research (WUNDeR) group, consisted of *T*_2_-weighted images of preterm infants (born <30 weeks' gestation) acquired shortly after birth (*n* = 12), preterm infants acquired at term-equivalent age (*n* = 12), and healthy term-born infants (born ≥38 weeks' gestation) acquired within the first 9 days of life (*n* = 12). For the NeoBrainS12 dataset, mean Dice scores comparing MANTiS with manual segmentations were all above 0.7, except for the cortical gray matter for coronal images acquired at 30 weeks. This demonstrates that MANTiS' performance is competitive with existing techniques. For the WUNDeR dataset, mean Dice scores comparing MANTiS with manually edited segmentations demonstrated good agreement, where all scores were above 0.75, except for the hippocampus and amygdala. The results show that MANTiS is able to segment neonatal brain tissues well, even in images that have brain abnormalities common in preterm infants. MANTiS is available for download as an SPM toolbox from http://developmentalimagingmcri.github.io/mantis.

## Introduction

Brain development during the neonatal period is an important determinant for a range of neurodevelopmental outcomes in childhood and adolescence. Being born preterm (<37 weeks' gestation) can lead to deviations in typical brain development that may have consequences for multiple neurodevelopmental domains, including cognition (for example IQ, attention, processing speed, memory, learning, and language; Anderson, 2014), movement (Williams et al., 2010), behavior (Hutchinson et al., 2013), and visual perception (Molloy et al., 2013). Characterizing and quantifying differences in brain developmental trajectories associated with preterm birth is important for generating biomarkers of neurodevelopmental outcomes and for evaluating the efficacy of interventions to improve long-term outcomes for infants born preterm (Anderson et al., 2015; Van Horn and Pelphrey, 2015). Magnetic resonance (MR) imaging is a vital tool allowing *in-vivo* measurement of many aspects of brain structure, such as the distribution of brain tissue types (tissue classification). Tissue classification enables volumetric studies to provide quantitative measures that are pivotal for studying brain injury and altered development associated with preterm birth, as previously demonstrated (Huppi et al., 1998; Inder et al., 2005; Thompson et al., 2007; Cheong et al., 2013). Tissue classification is also a precursor to many other analytic approaches, such as cortical parcellation, cortical thickness measurement and structural connectivity, and can provide seeds and masks for tractography and functional MR imaging studies.

Brain tissue classification methods require some form of prior information and a mechanism to adapt the prior information to the novel data, i.e., the MR image being classified. The prior information can take a variety of forms, including tissue intensity distributions and expected spatial distributions (Cocosco et al., 2003; Ashburner and Friston, 2005; Xue et al., 2007; Anbeek et al., 2008, 2013; Shi et al., 2010, 2011; Avants et al., 2011; Shiee et al., 2011) provided by atlases or manually segmented examples, or structural information encoded via the combination of morphological filtering and segmentation steps (Gui et al., 2012). Probabilistic atlases, representing tissue class distributions for a population, can provide a compact and convenient form of prior that can be used for stable and accurate segmentation if the novel data are not too different. Adaptation of prior information to novel data may occur via the classification process, registration, or a combination of both. Compared with automated methods for tissue classification in MR images of adults, there are a number of significant differences and challenges in neonatal MR images that necessitate the development of specialized, neonatal-specific methods for tissue classification. Neonatal MR images typically exhibit lower signal to noise ratios, larger voxels relative to brain size (leading to increased partial voxel effects), variable intensities within tissue classes, and differing between-tissue intensity contrasts compared with adults due to the incomplete myelination of the neonatal brain (Huppi et al., 1998; Lodygensky et al., 2010; Heemskerk et al., 2013). Limited myelination results in a reversal of relative gray matter and white matter intensities on *T*_1_- and *T*_2_-weighted scans of neonates (Holland et al., 1986). In addition, neonatal scans tend to exhibit high variability in brain shape and pathology, particularly in the context of preterm birth. For example, enlarged, distorted ventricles and hyper- and hypo-intense regions are common in preterm brain injury (Kidokoro et al., 2013). Collectively, these factors provide significant challenges for adapting approaches used in adult MR images to neonates.

There is a range of methods used for tissue classification in adult MR imaging studies, and key components of many have been adapted and extended for use in analysis of neonatal images. Current literature, discussed below, describes methods for *T*_1_-, *T*_2_-, both *T*_1_- and *T*_2_-weighted, and diffusion weighted scans, and addresses a range of issues experienced with neonatal MR imaging data. Earlier work focused on data-driven approaches to tissue classification. However, the increasing availability of neonatal atlases has resulted in more atlas-based methods, with advances focusing on effective use of atlases in the presence of high natural variability.

It is of practical importance to note the steps required in order for a method used in adult MR images to be applied to a neonatal dataset. For example, methods that use supervised classification are likely to be applicable to many types of image data, however these methods may require segmented examples in order to train the classifiers, making it potentially more difficult to apply such methods to novel neonatal data. Techniques utilizing tissue probability maps can be quite robust for a range of acquisition types for brains without pathology, as is evident from the widespread use of tools such as Unified Segmentation (Ashburner and Friston, 2005) in adult studies. However methods such as Unified Segmentation are likely to require new tissue probability maps for very different populations, such as neonates.

Methods for neonatal brain tissue classification proposed by Anbeek et al. (2008, 2013), Chita et al. (2013), and Srhoj-Egekher et al. (2013) employed supervised classification using non-parametric classifiers such as K nearest neighbor (KNN) classifiers. The strategies adopted can vary according to the form of features utilized by the classifiers, and the way in which sample distributions are generated. For example, Anbeek et al. (2008, 2013) employed supervised classification using manually segmented examples, with intensity and position information as features. Chita et al. (2013) also employed manual segmentations but added textural features. Srhoj-Egekher et al. (2013) used KNN to refine initial priors derived from a multi atlas-based segmentation.

Other neonatal brain tissue classification methods employed Expectation Maximization (EM) or variants of this framework, such as those by Xue et al. (2007), Shiee et al. (2011), Shi et al. (2010, 2011), Habas et al. (2010), Cardoso et al. (2013), and Avants et al. (2011). The EM is a general-purpose technique that has been widely applied in brain tissue classification, especially in conjunction with prior information in the form of probabilistic atlases and neighborhood constraints. Habas et al. (2010) demonstrated classification of fetal MR images using these approaches. Xue et al. (2007) employed EM for cortex classification, with extensions to deal with partial voxel effects. Shi et al. (2010, 2011) employed EM to segment neonatal MR images based on subsequently acquired longitudinal data. Shiee et al. (2011) and Cardoso et al. (2013) extended the standard EM approach to improve adaptive properties when the atlas does not approximate the unique data well, such as in pathologies associated with preterm birth; without such modifications the segmentation results can be biased toward the template. An alternative approach by Weisenfeld involves registering a library of manually segmented examples and classifying via an EM algorithm for simultaneous truth and performance evaluation (STAPLE) (Weisenfeld and Warfield, 2009). Prastawa et al. (2005) employed KNN in atlas creation, followed by robust graph techniques to estimate intensity distributions based on the atlas and EM coupled inhomogeneity correction and classification.

Brain tissue classification methods employing prior information in the form of atlases or manual segmentation typically require registration to align new image data, with methods not employing any prior spatial information being the exception. With the exception of Shi et al. (2010), all previous methods for neonatal brain tissue classification employed separate registration steps, rather than combining registration and tissue classification. The quality of registration required for successful tissue classification is likely to be strongly dependent on the segmentation algorithm, and registration steps can introduce additional complexity when tuning segmentation algorithms for novel data. Coupling the registration and tissue classification can improve the reliability of both, which may reduce the level of user intervention required for novel data. The best known combined tissue classification and registration for analysis of adult MR images is the “Unified Segmentation” algorithm of Ashburner and Friston (2005). Unified segmentation is a generative model combining tissue classification, bias inhomogeneity correction and nonlinear registration to a template in an EM framework. However, the unified segmentation method does not provide sufficient adaptability to successfully segment neonatal MR images, particularly those with brain abnormalities common in preterm infants, such as enlarged ventricles. There are several factors contributing to this limited adaptability in the context of neonatal MR images. For example, large differences between an atlas and the image being classified can lead to large errors in the initial estimate of tissue intensity distributions and require large deformations from the registration phase. The registration used by the unified segmentation algorithm incorporates a relatively small number of parameters and may not represent large, nonlinear deformations.

An alternative neonatal brain tissue segmentation approach was recently proposed by Gui et al. (2012), in which prior (problem-specific) information is encoded in the structure of filtering morphological segmentation steps. Gui et al.'s approach is similar to traditional data-driven approaches used in domains like microscopy, involving filtering steps to enhance structures of interest at various stages, and seed-based segmentation steps, with construction of seeds informed by prior knowledge. Such data-driven approaches are attractive due to elimination of registration steps and templates, but may be challenging to make robust to varying image quality and acquisition protocol; i.e., they are very adaptable but may be difficult to develop and stabilize.

Recently the NeoBrainS12 challenge compared eight different brain tissue segmentation methods for *T*_1_- and *T*_2_-weighted MR images of preterm-born neonates (Isgum et al., 2015). The challenge compared automated methods with manual brain tissue segmentations for axial and coronal MR images of preterm infants acquired at 30 and 40 weeks' corrected gestational age. Of the eight methods included in the NeoBrainS12 challenge paper, the majority (Anbeek et al., 2008; Avants et al., 2011; Gui et al., 2012; Cardoso et al., 2013; Chita et al., 2013; Srhoj-Egekher et al., 2013) have been published separately, and were based on methods discussed above. The challenge found “that the participating methods were able to segment all tissue classes well, except myelinated white matter” (Isgum et al., 2015). Most of the methods participating in the NeoBrainS12 challenge are not publicly available, although some of the components used in the methods are available.

In this paper we introduce a freely available neonatal tissue segmentation technique, MANTiS (Morphologically Adaptive Neonatal Tissue Segmentation), which utilizes a combination of unified segmentation, template adaptation via morphological segmentation tools and topological filtering (described next). This unique combination of techniques aims to provide adaptability from the morphological segmentation and filtering, while retaining stability from the unified segmentation and neonatal atlas. The addition of the morphological segmentation step provides fast, reliable, data-driven adaptability for neonatal MR images that have unusual anatomy, particularly enlarged ventricles, which are common in preterm infants. The morphological filtering step removes unusual image contrasts that cause incorrect classification, without changing desirable interclass boundaries. MANTiS utilizes a 40-week infant template (Kuklisova-Murgasova et al., 2011) and segments the brain into eight regions or tissue types: cortical gray matter, white matter, deep nuclear gray matter, cerebellum, brainstem, cerebrospinal fluid (CSF), hippocampus, and amygdala. The structure we propose allows the data-driven phases to exploit information derived from atlas-based priors, considerably simplifying their design. We are also able to utilize an established and tested tool for adult brain tissue classification, unified segmentation, without modification (Ashburner and Friston, 2005). We validate the proposed method using two independent datasets: manual segmentations of preterm neonatal MR images from the NeoBrainS12 challenge, and manually edited segmentations of preterm *and term* neonatal MR images from the Washington University NeuroDevelopmental Research (WUNDeR) group.

The key novel steps of MANTiS that provide adaptability use the following morphological image processing tools:

Morphological segmentation can be performed via morphological watershed transform from markers, a technique for image segmentation based on geophysical principles. Various forms of the watershed transform exist and have been used in neuroimaging (Hahn and Peitgen, 2000; Gui et al., 2012; Beare et al., 2013). The segmentation approach considers the image as a terrain, with higher intensity corresponding to higher altitude. Water is allowed to flood the terrain from several points, typically indicated by a marker or seed image. Each seed will result in a separate region in the final segmentation. Region boundaries occur where water from different flooding regions meet, which occurs at high intensity “ridge lines” in the terrain. The development of segmentation procedures thus becomes a process of transforming an image to create a “control image” so that high intensity ridges occur where boundaries are desired (e.g., by edge detection), generating markers and applying a watershed transform (Beucher and Meyer, 1992). The design of the control and seed image generation is application-specific, while the watershed transform segmentation step is parameter-free.

**Figure 1:**
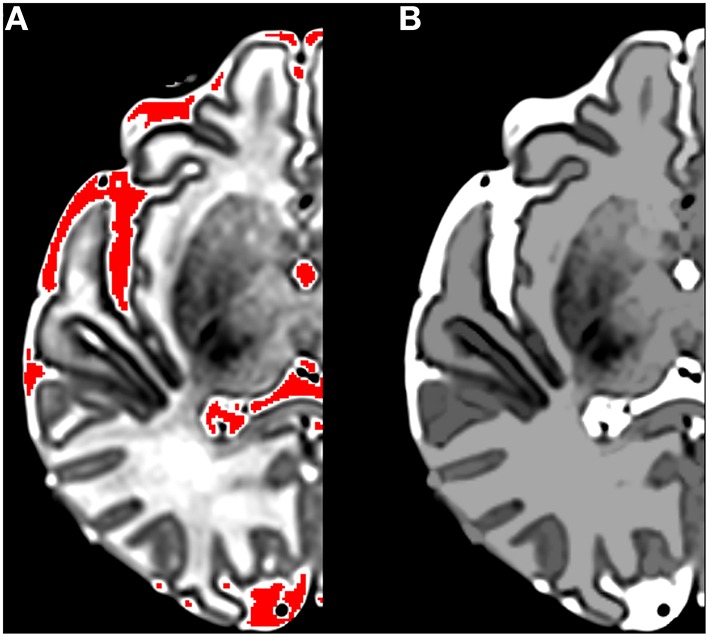
**The results of applying a reconstruction by dilation to a single slice of a brain scan using the highest intensity regions as markers**. **(A)** Shows in red the marker regions used for the reconstruction; **(B)** Shows the post-reconstruction image. Note that the intensity of cerebrospinal fluid (CSF) and gray matter remains constant while the intensity of isolated high intensity white matter regions is reduced and edges are preserved.

Morphological filtering can be performed via reconstruction by dilation, which is a filtering process that operates on grayscale connected components to remove some image features without changing others. Morphological reconstruction is different to the better-known morphological erosion/dilation using structuring elements. For example, reconstruction by dilation can be used to remove isolated high intensity spots while leaving low intensity spots and edges unchanged—it is sensitive to the topology of the image rather than the size of image features. Reconstruction by dilation can be implemented using an iterative application of unit dilations and masking. The process begins with a marker image (typically a subset of the original image) and a mask image (the image being filtered). In the limiting case, the marker may be the single highest intensity voxel in the image. The marker image is dilated and then masked by the mask image using a voxel-wise minimum operation. In the example of a single voxel seed, the dilation step enlarges the seed while the masking step restricts the intensity of the enlarged region to that of the mask. The steps are iterated until stability is reached. In the case of a single voxel seed, the dilation steps will result in values being propagated over the entire image. The result is that each voxel in the output image is replaced by the lowest value on the highest intensity path connecting that voxel to the marker. Thus, a high intensity spot will be deleted while contours of a high intensity region containing the marker will be retained. Figure 1 illustrates the results of applying a reconstruction by dilation to a single slice of a brain scan using the highest intensity regions as markers. Contours between tissue classes are preserved after filtering, while isolated high intensity regions are removed.

## Methods

### Pre-processing

The input for the MANTiS tissue classification pipeline is a brain-extracted *T*_2_-weighted image. The brain extraction can be done using the Brain Extraction Tool (BET) within the Functional MR Imaging of the Brain Software Library (FSL) (Smith, 2002). The results in the current study were obtained using BET for brain extraction. Alternatively, there is a preliminary version of a brain extraction tool available within the MANTiS toolbox, which can be used prior to the MANTiS tissue classification pipeline.

### MANTiS processing pipeline

Processing consists of several phases, as detailed below. Identical processing is applied to all images, irrespective of anatomy or gestational age, and no parameter selection is required. Thus, the processing pipeline requires no prior information about subject type. All processing phases are performed without manual intervention.

#### Phase 1. initial tissue classification

Initial classification uses the unified segmentation approach implemented in the Statistical Parametric Mapping (SPM) software and a neonatal template. This pipeline uses the “New Segment” tool available in SPM version 8. The template used is a 40-week infant template developed by the Biomedical Image Analysis Group, Imperial College London (Kuklisova-Murgasova et al., 2011). This probabilistic atlas includes six regions: cortical gray matter, subcortical gray matter, white matter, CSF, brainstem and cerebellum. A modification was made to this template's subcortical gray matter label to separate out the hippocampus, amygdala and deep nuclear gray matter. Deep nuclear gray matter was defined as any tissue superior to a border placed at the most inferior point of the putamen once the amygdala was first visualized. Hippocampus and amygdala were separated as previously described (Thompson et al., 2008, 2012), with reference to neuroanatomical atlases (Duvernoy, 1988; Entis et al., 2012).

**Figure 2:**
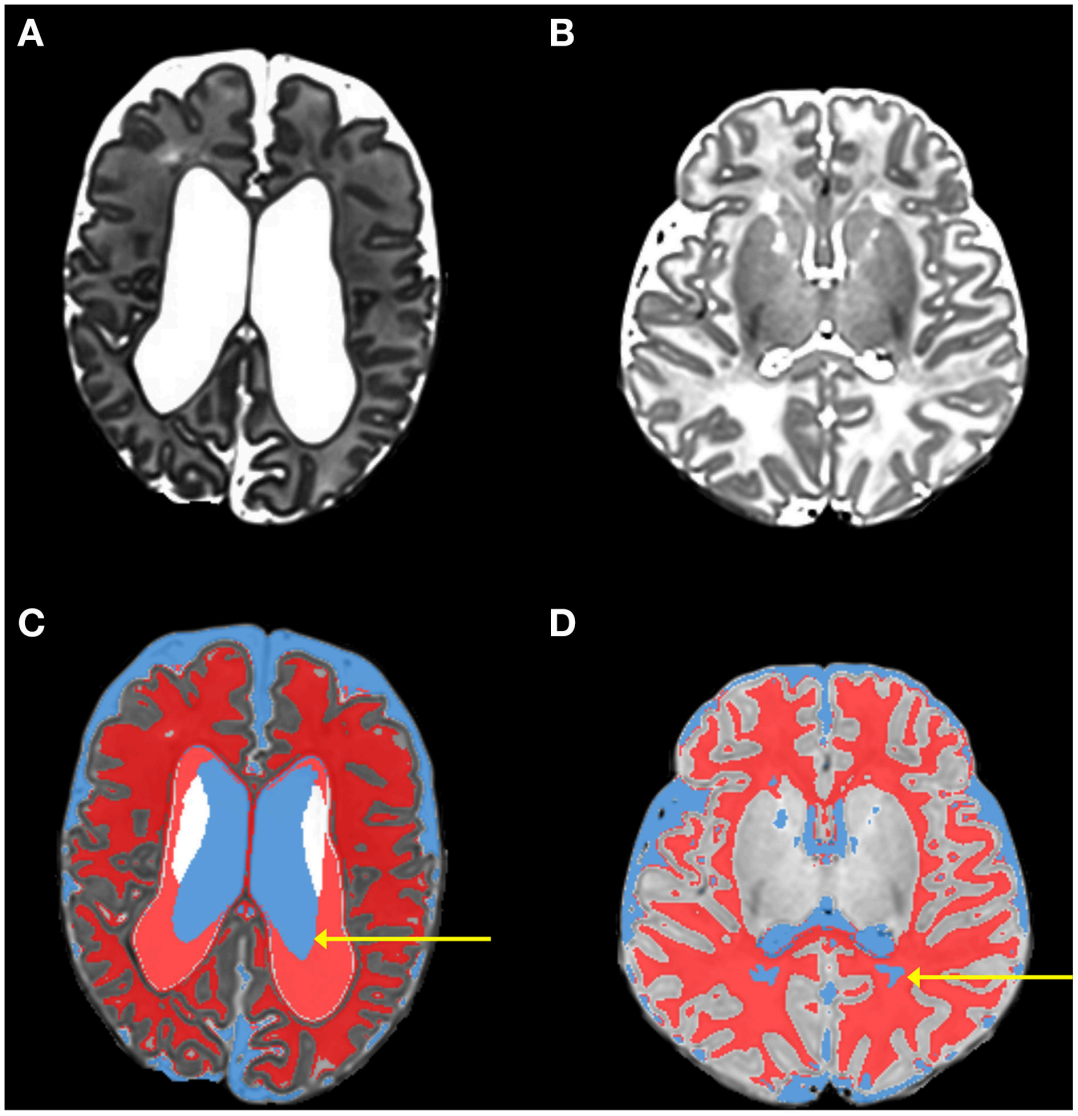
**Images from phase 1 (initial classification using unified segmentation and the neonatal template)**. This figure shows original *T*_2_-weighted images of two infants [(**A,B**; both are preterm infants scanned at term-equivalent age] in comparison with the initial tissue classifications produced by phase 1 for the same infants [**(C,D)** respectively]. Typical errors that occur during the initial classification can be seen; in **(C)** the arrow highlights undersized ventricle (blue) classification coupled with excess white matter (red) classification; in **(D)** the arrow highlights white matter that has been misclassified as CSF.

During this initial phase all of the eight tissue types (cortical gray matter, deep nuclear gray matter, white matter, CSF, brainstem, cerebellum, hippocampus, and amygdala) are segmented, however there are often errors in the initial classification, which are subsequently corrected in the following phases. Errors in the initial classification occur when ventricles are enlarged and when white matter has high water content and therefore signal intensity similar to CSF (Figure 2). When the ventricles are large the classification of ventricles is undersized, with misclassification of CSF as white matter. The cause of the undersized ventricular segmentation is a combination of a number of factors that result from the adult template being poorly matched to the infant image being classified (as described in the Introduction). Peripheral CSF is typically correctly classified. A number of parameters, relating to number of Gaussians used in mixture models and severity of bias inhomogeneity, are available to control “New Segment.” The default parameter settings in MANTiS, used for all tests, are: 2 Gaussians for each tissue class, very light bias regularization (0.0001) and 60 mm bias FWHM.

#### Phase 2. morphological segmentation

**Figure 3:**
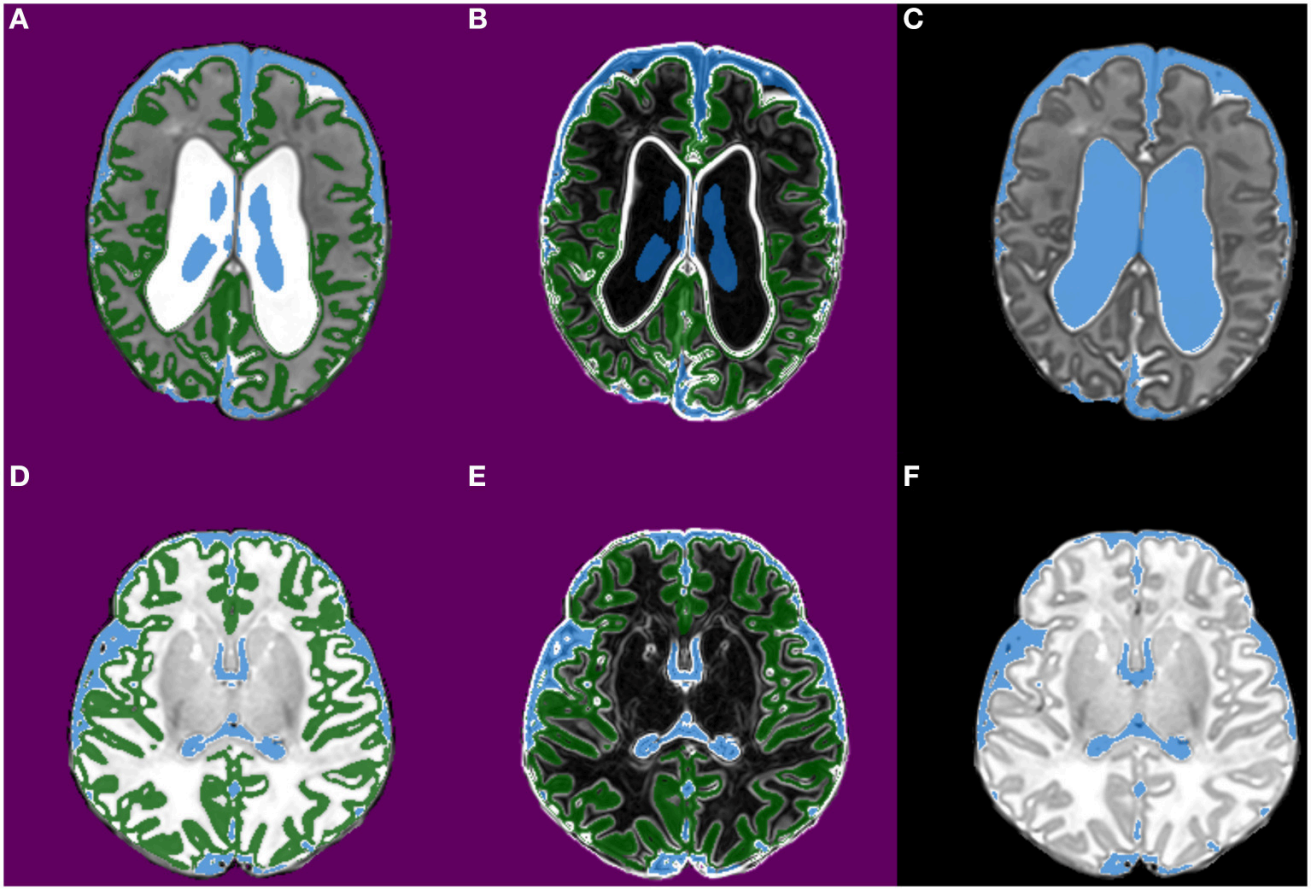
**Images from phase 2 (morphological segmentation using watershed transform from markers)**. The images are from two infants [the top row is the first infant and the bottom row is the second infant; both are preterm infants scanned at term-equivalent age]. **(A,D)** Show the three markers used for watershed segmentation of CSF; CSF (shown in blue), cortical gray matter (green), and background (purple). **(B,E)** Show the corresponding gradient images (the terrain or control image); strong edges in the gradient images become boundaries in the watershed segmentation. **(C,F)** Show the CSF segmentation produced in this phase.

Morphological segmentation, in the form of a morphological watershed transform from markers, is used to develop a reliable segmentation of the ventricles (Figure 3). The key properties of the watershed segmentation are that it does not change the size of “typical” ventricle segmentations, but appropriately enlarges undersized segmentations. The morphological watershed transform requires a set of markers (one marker for each class being segmented) and a control image. The marker for CSF is constructed by thresholding the phase 1 CSF probability map at a level of 0.9 and removing connected components smaller than 500 mm^3^. The cortical gray matter marker is created by thresholding the phase 1 cortical gray matter probability map at a level of 0.7. The output non-brain voxels defined during brain extraction are used to define a background marker. The control image is generated by applying a simple multiscale gradient filter, using Gaussian convolution kernels with σ = 0.25 mm and σ = minimum voxel dimension. These parameters were empirically selected based on typical image resolutions and used for all subjects. The ventricle segmentation is then used to create a subject-specific template as follows: (1) The prior probability of CSF is adjusted by taking the maximum of the watershed ventricle segmentation and the original CSF probability map; (2) The template is warped to the estimate of subject space using the transformations estimated by phase 1, allowing subsequent stages to refine the transformation without having to re-estimate it.

#### Phase 3. morphological filtering

**Figure 4:**
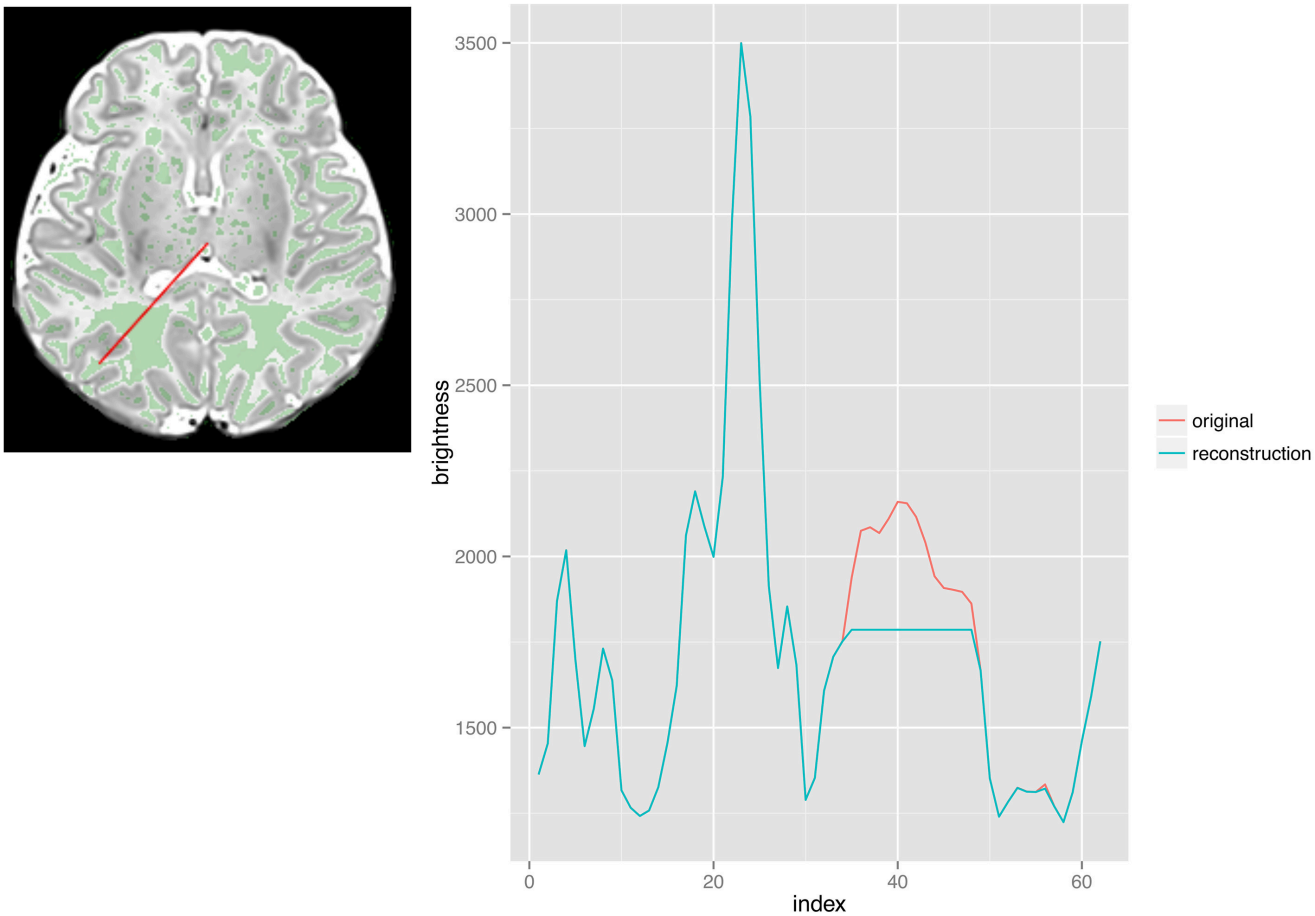
**Images from phase 3 (morphological filtering via reconstruction by dilation)**. This Figure illustrates the effect of reconstruction by dilation. The plot on the right is a profile along the red line in the image, in the medial to lateral direction. The green areas in the image correspond to places where the original is larger than the reconstruction, such as the red area in the plot.

Morphological filtering, in the form of a reconstruction by dilation, is used to eliminate the high intensity white matter regions that are misclassified as CSF. A reconstruction by dilation (described in the Introduction) can be used to remove isolated regions that are higher in intensity than their surroundings, such as peripheral white matter that has signal intensity similar to CSF. To achieve this, we use the CSF detected by phase 1 as the seed and the *T*_2_-weighted image as the mask image. In *T*_2_-weighted images, CSF intensity is greater than that of the white matter, which is greater than that of the gray matter. Seeding the reconstruction process using a subset of the highest intensity tissue type allows us to reduce the intensities of areas of white matter that are higher in intensity than their surroundings (Figure 4).

#### Phase 4. second classification using adapted template

A second classification is performed, again using the unified segmentation approach. In this version the subject-specific template from phase 2 is used in conjunction with the morphologically reconstructed *T*_2_-weighted image from phase 3.

#### Phase 5. final clean up and segmentation

**Figure 5:**
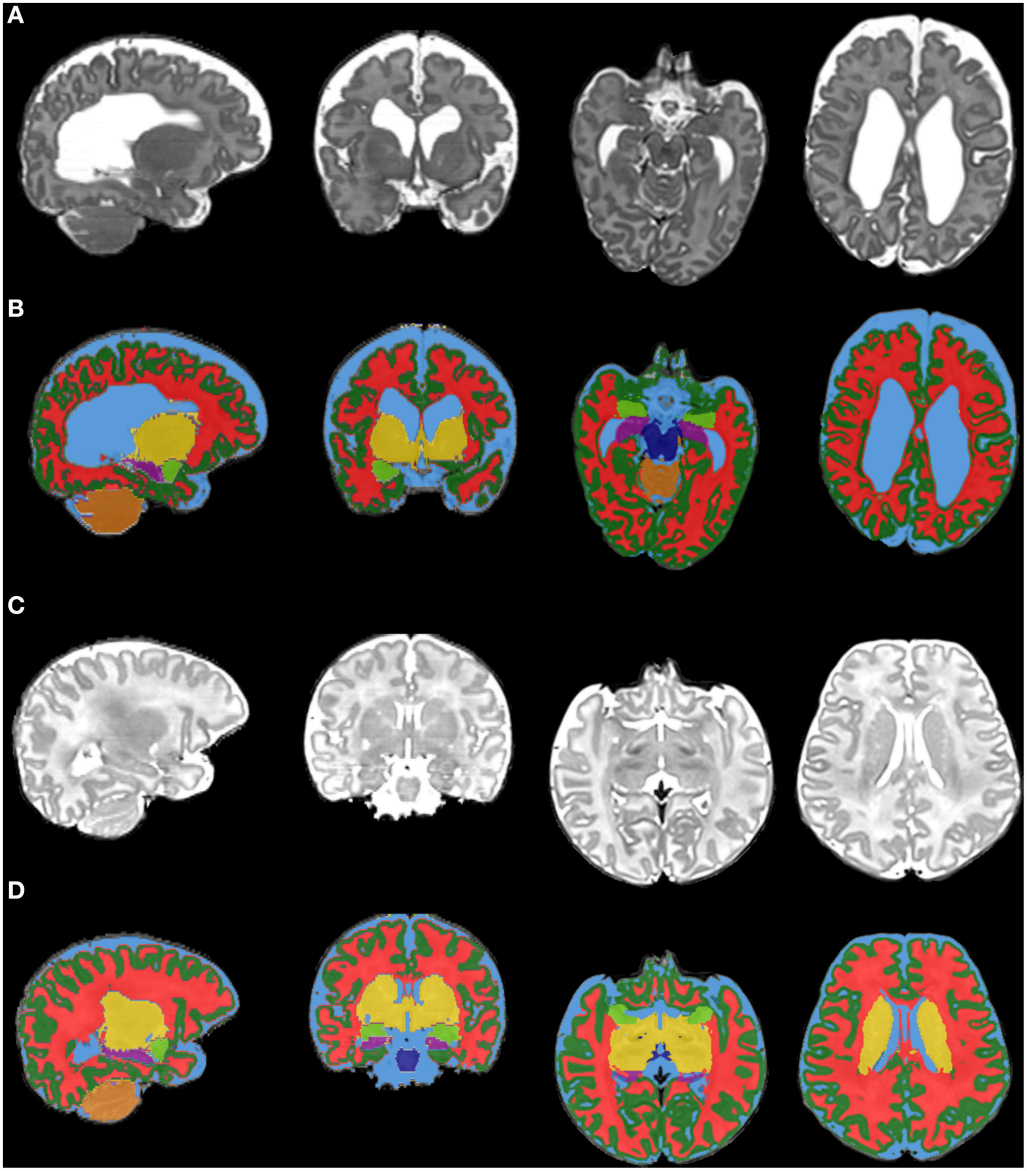
**Final tissue classification**. This figure shows the final tissue classification [rows **(B)** and **(D)**] in comparison with the original *T*_2_-weighted images [rows **(A,C)**]. The images are selected brain slices from two infants [first infant = rows **(A,B)**; second infant = rows **(C,D)**; both infants are preterm infants scanned at term-equivalent age]. The colors represent brain tissues/structures as follows: dark green, cortical gray matter; red, white matter; yellow, deep nuclear gray matter; light blue, CSF; dark blue, brainstem; orange, cerebellum; purple, hippocampus; light green, amygdala.

Isolated pockets of peripheral CSF that were removed by phase 3 are restored; recall that isolated high intensity regions are set to the intensity of the surrounding tissue, thus isolated pockets of CSF are set to the intensity of the surrounding gray matter. These pockets were correctly classified in phase 1, and can thus be detected by taking the difference between the gray matter classifications produced in phase 4 and phase 1. Isolated pockets of white matter are restored using the same approach. Figure 5 shows examples of the final tissue classification.

### Implementation

MANTiS is implemented as a toolbox for SPM. Morphological segmentation and filtering are implemented using components from the Insight Toolkit (ITK) (Yoo et al., 2002), www.itk.org. The MANTiS package for SPM versions 8 and 12 is available for download from http://developmentalimagingmcri.github.io/mantis.

### Computation time

Excluding pre-processing (brain extraction), the MANTiS pipeline requires approximately 7 min total processing time per 90 × 144 × 192 image (running on an Intel i7-4770 workstation with a 3.40 GHz processor, 4 cores (8 threads) and 32 GB RAM, through MATLAB version R2010b). Each round of unified segmentation (i.e., phase 1 and phase 4 described above- the initial and second classifications) takes about 3 min. Morphological segmentation and filtering (phase 2 and 3 above) take 5 s each, while construction of a subject-specific template (required for phase 4) takes 30 s and the final clean up (phase 5) takes 10 s.

### Validation

To evaluate the performance of MANTiS, we applied MANTiS to two independent image datasets, described below, for which manual segmentations or manually edited segmentations were available for comparison.

#### Participants in the validation datasets

The first dataset was provided by the NeoBrainS12 challenge and consisted of MR images from 3 groups of infants: axial MR images of preterm infants (born ≤30 weeks' gestation) acquired at 40 weeks' corrected gestational age (*n* = 5); coronal MR images of preterm infants acquired at 40 weeks' corrected gestational age (*n* = 5); coronal MR images of preterm infants acquired at 30 weeks' corrected gestational age (*n* = 5). No brain pathology was visible on the scans, and all infants obtained a normal score for the Griffiths Mental development scale when followed-up at age 15 months corrected for gestational age (Isgum et al., 2015). Full details of the NeoBrainS12 challenge are available at http://neobrains12.isi.uu.nl and in the corresponding publication (Isgum et al., 2015).

The second dataset, provided by the Washington University NeuroDevelopmental Research (WUNDeR) group, included MR images from three groups of infants: preterm infants (born <30 weeks' gestation) scanned at 27–32 weeks' gestational age (*n* = 12); preterm infants (born <30 weeks' gestation) scanned at term-equivalent age (37–41 weeks' gestational age, *n* = 12); healthy term-born infants (born ≥38 weeks' gestation, *n* = 12) scanned within the first 9 days of life. Thus, there were 36 different infants in total in the WUNDeR dataset. The infants were selected from a larger prospective, longitudinal study of infants recruited from St. Louis Children's Hospital. Inclusion criteria for the term-born infants included no maternal history of major medical or psychiatric illness, no maternal medication treatment or substance abuse history during the pregnancy, prenatal care (>5 visits), 5-min APGAR score ≥ 8, age-appropriate birth weight and head circumference, no admission to a neonatal or special care nursing unit and no documented neurological abnormality (no antenatal cerebral abnormality detected by fetal ultrasound, no concerns for chromosomal abnormality or congenital/acquired infection and no neonatal encephalopathy). All procedures were approved by the Washington University School of Medicine's Institutional Review Board. Informed, written consent for the study was obtained from parents or legal guardians for all participants.

#### MR image acquisition

The NeoBrainS12 images were acquired using a 3T Philips system as follows: (1) For the group of axial *T*_2_-weighted images acquired at 40 weeks, repetition time (TR) 6293 ms, echo time (TE) 120 ms, consecutive sections with thickness 2.0 mm, in-plane resolution 0.35 × 0.35 mm; (2) For the group of coronal *T*_2_-weighted images acquired at 40 weeks, TR 4847 ms, TE 150 ms, consecutive sections with thickness 1.2 mm, in-plane resolution 0.35 × 0.35 mm; (3) For the group of coronal *T*_2_-weighted images acquired at 30 weeks, TR 10085 ms, TE 120 ms, consecutive sections with thickness 2.0 mm, in-plane resolution 0.34 × 0.34 mm. *T*_1_-weighted images were also available for the challenge, but were not used by MANTiS. Additionally, two axial images acquired at 40 weeks' corrected gestational age and two coronal images acquired at 30 weeks' corrected gestational age, along with corresponding manual segmentations, were made available as training data. Mean (standard deviation) gestational ages at birth for the participants in the NeoBrainS12 challenge were as follows: (1) Axial images acquired at 40 weeks' corrected gestational age, 28.3 (2.3) weeks; (2) Coronal images acquired at 30 weeks' corrected gestational age, 26.4 (1.2) weeks; (3) Coronal images acquired at 40 weeks' corrected gestational age, 27.0 (0.9) weeks. Other participant characteristics for the NeoBrainS12 dataset have not been made available.

All infants in the WUNDeR dataset were scanned without the use of sedating medications. Noise protection during scanning was provided by earmuffs (Natus Medical, Foster City, CA). Arterial oxygen saturation and heart rate were continuously monitored throughout the acquisition. A Neonatal Intensive Care Unit (NICU) staff member was present in the scanner room throughout the study. MR imaging was performed using a Siemens (Erlangen, Germany) 3T Trio scanner using an infant-specific, quadrature head coil (Advanced Imaging Research, Cleveland, OH). *T*_2_-weighted images were acquired using a turbo spin echo (TSE) sequence (TR/TE 8600/161 ms, voxel size 1 × 1 × 1 mm^3^). Participants were excluded if their *T*_2_-weighted image had significant movement artifact. Images were systematically assessed for the presence of cerebral injury by a certified pediatric neurologist (C.D.S.) and neonatologist (T.E.I.) at St. Louis Children's Hospital (Kidokoro et al., 2014). Three of the preterm infants had grade 2 intraventricular hemorrhage (IVH), but none had grade 3/4 IVH. Four preterm infants had grade 1–2 (mild-moderate) cerebellar hemorrhage and one preterm infant had grade 3 (severe) cerebellar hemorrhage. No infants had cystic periventricular leukomalacia (PVL). Participant characteristics of the WUNDeR dataset are shown in Table 1.

**Table 1:** **Participant characteristics of the WUNDeR dataset**.

	**Preterm (scanned at 27–32 weeks), *n* = 12**	**Preterm (scanned at TEA), *n* = 12**	**Term, *n* = 12**
GA at birth (weeks)	27.0 (23–29)	26.4 (23–30)	39.4 (38–41)
GA at scan (weeks)	29.6 (27–32)	37.9 (37–41)	39.5 (38–41)
Birth weight (g)	1006 (670–1400)	911 (700–1410)	3260 (2585–4360)
Male, n (%)	7 (58)	4 (33)	7 (58)

*Data are means (minimum–maximum value) unless otherwise stated. GA, gestational age; TEA, term-equivalent age*.

#### Manual segmentations for the NeoBrainS12 dataset

The manual segmentation of the NeoBrainS12 images was carried out using T_2_-weighted images and in-house software, “either by MDs who were working toward a PhD in neonatology, or by trained medical students. The segmentations were verified independently by three neonatologists with each at least seven years of experience in reading neonatal MRI scans” (Isgum et al., 2015). Details of the manual segmentation protocol are available at http://neobrains12.isi.uu.nl/reference.php and in the NeoBrainS12 publication (Isgum et al., 2015). Eight tissue classes were manually delineated; the cortical gray matter (including hippocampus and amygdala), myelinated white matter, unmyelinated white matter, deep nuclear gray matter (i.e., basal ganglia and thalami), brainstem, cerebellum, CSF in the ventricles and CSF in the extracerebral space. For the purpose of comparison with the MANTiS segmentation, myelinated and unmyelinated white matter classes were combined, and the two CSF classes were combined.

#### Manually edited segmentations for the WUNDeR dataset

Both *T*_1_ and *T*_2_-weighted images were used to generate initial segmentations using the Atropos segmentation algorithm (Avants et al., 2011) within the Advanced Normalization Tools (ANTs) software package to create cortical gray matter, white matter, subcortical gray matter, CSF and cerebellar volumes. This methodology was similar to “Method D” in the NeoBrainS12 challenge (Isgum et al., 2015). The segmentations were then extensively manually corrected by analysts trained in neonatal neuroanatomy using the ITK-SNAP image software package (Yushkevich et al., 2006), the brainstem class was added, and the subcortical gray matter was separated into hippocampus, amygdala and deep nuclear gray matter. One individual reviewed all segmentations and provided feedback for further editing, as needed, to minimize inter-rater inconsistencies.

#### MANTiS segmentation

The *T*_2_-weighted images from the NeoBrainS12 and WUNDeR datasets were automatically segmented using the MANTiS pipeline as described above. For comparison with the NeoBrainS12 segmentations, hippocampus and amygdala were combined with the cortical gray matter class. Identical processing was applied to all subjects in the NeoBrainS12 dataset and the WUNDeR dataset. No manual intervention or parameter selection was required.

#### Comparison between MANTiS and manual or manually edited segmentations

Dice scores, mean surface distances and Hausdorff distances were used to compare MANTiS segmentation results with manual segmentations and manually edited segmentations for the NeoBrainS12 and WUNDeR datasets respectively. Dice scores indicate the proportion of overlap between the MANTiS segmentation and the manual/manually edited segmentation, and thus tend to be high for regions with large volume and low for small regions. The mean surface distance is the average distance between segmentation boundaries, in millimeters, and is thus less dependent on region volume. Hausdorff distance is also measured in millimeters, but corresponds to the largest distance between segmentation boundaries, and is thus a measure of how poor the least accurate part of the segmentation is. A large Hausdorff distance can be caused by a difference that leads to a minimal change in volume. For example a single incorrectly classified voxel in an unusual position can lead to a large Hausdorff distance, but would make minimal difference to volume estimates.

## Results

### Validation results using the NeoBrainS12 dataset

**Figure 6:**
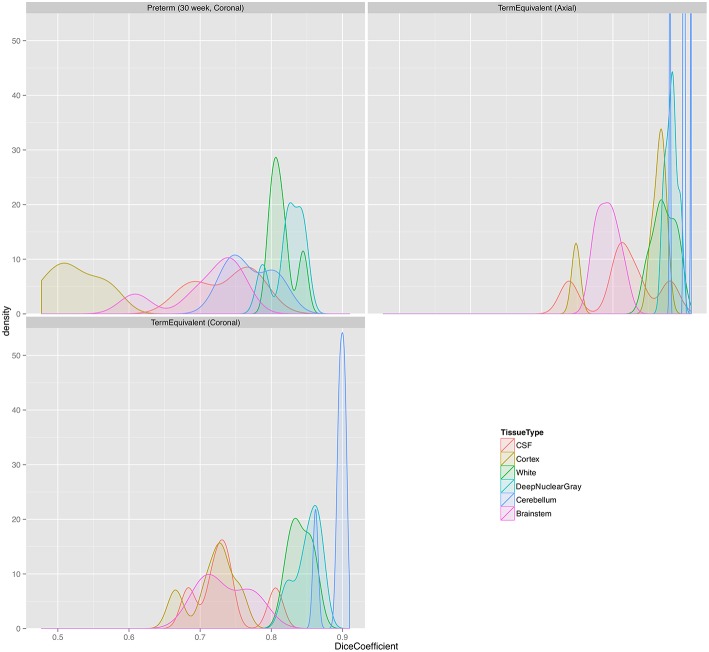
**Distribution plots of Dice scores for the NeoBrainS12 dataset**. Preterm (30 week, Coronal), group of coronal images of preterm infants acquired at 30 weeks' corrected gestational age; TermEquivalent (Axial), group of axial images of preterm infants acquired at 40 weeks' corrected gestational age; TermEquivalent (Coronal), group of coronal images of preterm infants acquired at 40 weeks' corrected gestational age; CSF, cerebrospinal fluid.

Table 2 shows Dice scores, mean surface distances and Hausdorff distances for comparison between MANTiS segmentations and manual segmentations for the NeoBrainS12 dataset. Results are given for the cortical gray matter, white matter, deep nuclear gray matter, cerebellum, CSF and brainstem, for each group of infants separately (i.e., axial images acquired at 40 weeks' corrected gestational age, coronal images acquired at 30 weeks' corrected gestational age and coronal images acquired at 40 weeks' corrected gestational age). Average scores across groups and across tissue types are also given. Distributions of the Dice scores are shown in Figure 6. In general, Dice scores were good; all were above 0.7 except for the cortical gray matter for the 30-week coronal images, which had the lowest Dice score of 0.52. In terms of the groups, the highest (best) Dice scores were obtained for the 40-week axial images (average 0.84 across all tissue types) and the lowest (worst) for the 30-week coronal images (average 0.73 across all tissue types). In terms of the tissue types, the best Dice scores were obtained for the cerebellum and deep nuclear gray matter (average 0.85 across all groups), and the worst for the cortical gray matter (average 0.69 across all groups). This result is similar to that of the NeoBrainS12 challenge, which found that the teams achieved the best Dice scores for the cerebellum and deep nucleur gray matter, likely due to the compact shape of these structures (Isgum et al., 2015). Mean surface distances were all approximately 1 mm or less, and Hausdorff distances were all approximately 25 mm or less. Given that the NeoBrainS12 dataset had anisotropic voxels in which the largest dimension was 1.2 or 2 mm, the mean surface distances obtained in the current study suggest that the MANTiS segmentation and manual segmentation boundaries were usually within one voxel of each other. The mean surface distances and Hausdorff distances were better for the 40-week images than the 30-week images. In terms of the tissue types, the best mean surface distances and Hausdorff distances were obtained for the cortical gray matter and the worst for the brainstem.

**Table 2:** **Dice scores, mean surface distances (MSD) and Hausdorff distances (HD) for MANTiS compared with manual segmentations for the NeoBrainS12 dataset**.

**Brain region/Tissue type**	**Preterm infants scanned at 40 weeks, axial scans**			**Preterm infants scanned at 30 weeks, coronal scans**			**Preterm infants scanned at 40 weeks, coronal scans**			**All infants**		
	**Dice, mean (SD)**	**MSD, mean**	**HD, mean**	**Dice, mean (SD)**	**MSD, mean**	**HD, mean**	**Dice, mean (SD)**	**MSD, mean**	**HD, mean**	**Dice, mean (SD)**	**MSD, mean**	**HD, mean**
Cortical gray matter	0.84 (0.05)	0.19	8.61	0.52 (0.04)	0.57	11.26	0.72 (0.03)	0.29	6.51	0.69 (0.14)	0.35	8.79
White matter	0.87 (0.02)	0.31	24.74	0.82 (0.02)	0.66	9.20	0.84 (0.02)	0.45	27.72	0.84 (0.03)	0.47	20.55
Deep nuclear gray matter	0.88 (0.01)	0.70	8.19	0.82 (0.02)	0.85	19.78	0.85 (0.02)	1.00	8.40	0.85 (0.03)	0.85	12.12
Cerebellum	0.90 (0.01)	0.57	8.55	0.77 (0.03)	0.97	41.76	0.89 (0.02)	0.66	9.68	0.85 (0.06)	0.73	20.00
CSF	0.77 (0.07)	0.35	9.12	0.74 (0.04)	0.41	8.69	0.74 (0.04)	0.48	9.10	0.75 (0.05)	0.41	8.97
Brainstem	0.79 (0.02)	0.69	24.77	0.71 (0.06)	1.04	25.56	0.73 (0.04)	0.84	18.23	0.74 (0.05)	0.86	22.85
Average	0.84 (0.03)	0.47	14.00	0.73 (0.04)	0.75	19.38	0.80 (0.03)	0.62	13.27			

*CSF, cerebrospinal fluid*.

**Figure 7:**
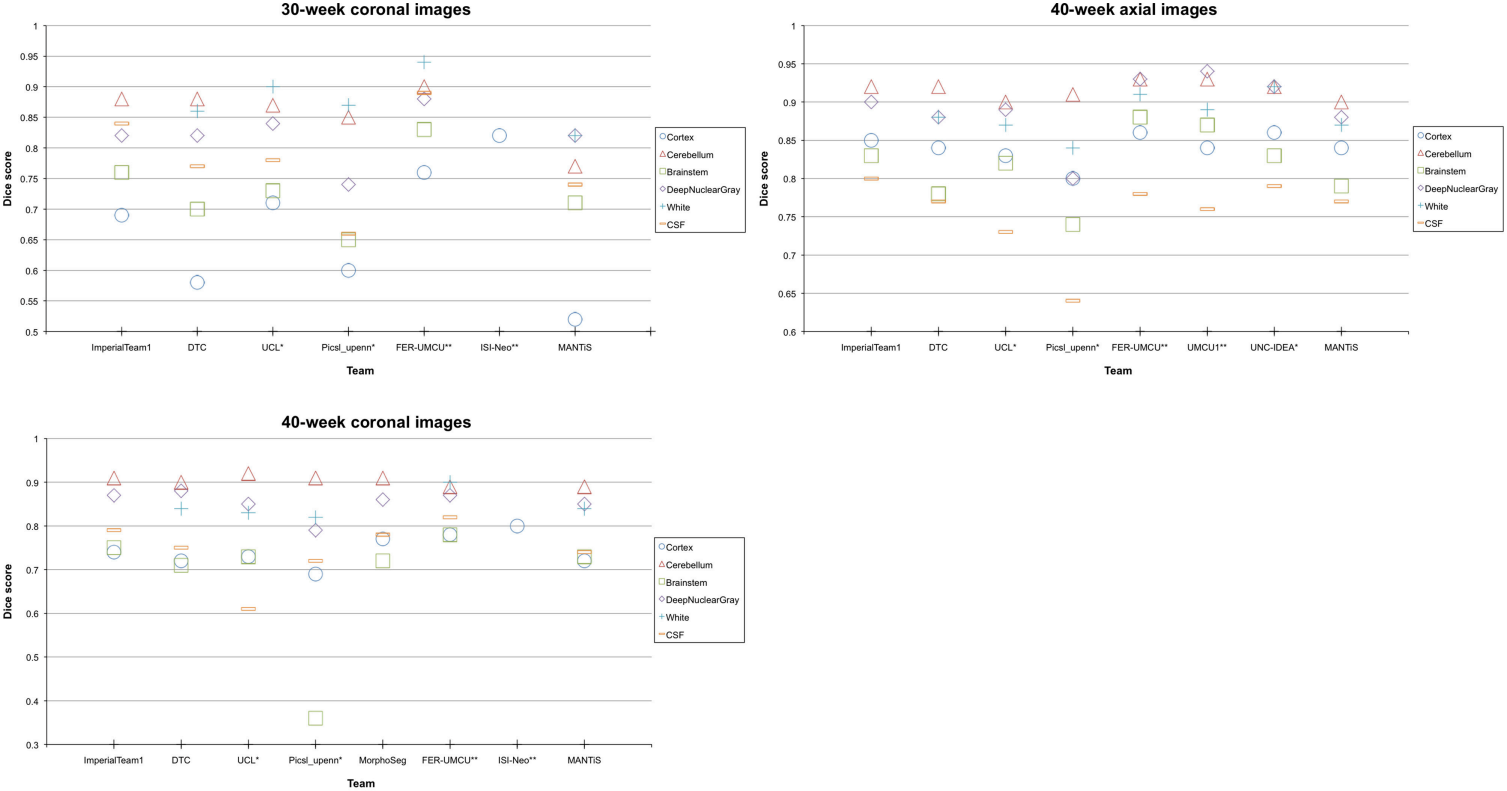
**Graphs showing MANTiS' mean Dice scores compared with mean Dice scores of the other methods that participated in the NeoBrainS12 challenge**. ImperialTeam1, Imperial College London, Method A; DTC, University of Oxford, Method B; UCL, University College London, Method C; Picsl_upenn, University of Pennsylvania, Method D; FER-UMCU, University of Zagreb and University Medical Center Utrecht, Method F; ISI-Neo, University Medical Center Utrecht, Method H; MorphoSeg, Geneva University Hospital, Method E; UMCU1, University Medical Center Utrecht, Method G; UNC-IDEA, University of North Carolina (IDEA lab), ^*^The results have been evaluated over three images initially available for the web-based challenge. ^**^The authors had more image and reference data available than provided by this challenge. For more information on the other methods that participated in the NeoBrainS12 challenge, please see the NeoBrainS12 publication (Isgum et al., 2015) or the NeoBrainS12 website (http://neobrains12.isi.uu.nl).

Additionally, MANTiS' Dice scores, mean surface distances and Hausdorff distances for the images acquired at 40 weeks were generally in the top 50% of the teams that participated in the NeoBrainS12 challenge, although MANTiS ranked slightly lower for the images acquired at 30 weeks. Figure 7 shows MANTiS' Dice scores alongside those of the other teams that participated in the NeoBrainS12 challenge. Results of the NeoBrainS12 challenge can also be seen online (http://neobrains12.isi.uu.nl/mainResults.php).

### Validation results using the wunder dataset

**Figure 8:**
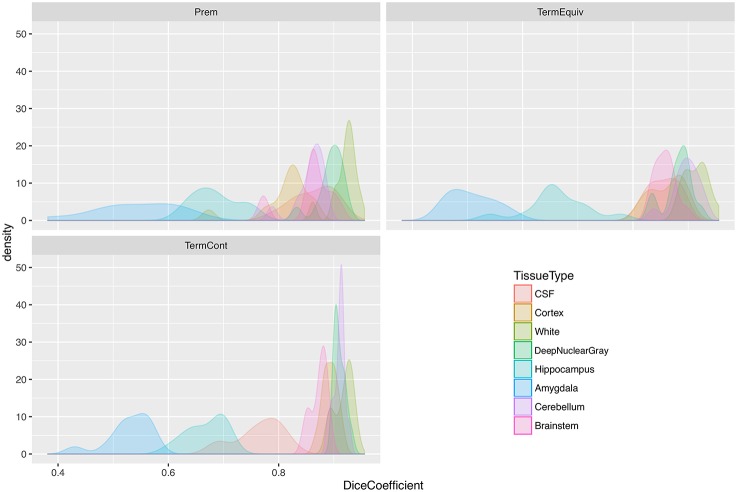
**Distribution plots of Dice scores for the WUNDeR dataset**. Prem, group of preterm infants scanned at 27–32 weeks' gestational age; TermEquiv, group of preterm infants scanned at term-equivalent age; TermCont, group of term-born infants; CSF, cerebrospinal fluid.

Table 3 shows Dice scores, mean surface distances and Hausdorff distances for comparison between MANTiS segmentations and manually edited segmentations for the WUNDeR dataset. Results are given for the cortical gray matter, white matter, deep nuclear gray matter, cerebellum, CSF, brainstem, hippocampus and amygdala, for each group of infants separately (i.e., preterm scanned at 27–32 weeks' gestational age, preterm scanned at term-equivalent age, and term-born infants). Average scores across groups and across tissue types are also given. Distributions of Dice scores for the three groups in the WUNDeR dataset are shown in Figure 8. In general, Dice scores were good; all were above 0.75, excluding the hippocampus and amygdala. Dice scores were very similar between the three groups (preterm, term-equivalent and term). In terms of the tissue types, the best Dice scores were obtained for the white matter (average 0.92 across all groups) and worst for the hippocampus and amygdala (average 0.67 and 0.53 respectively across all groups). Mean surface distances were all approximately 1 mm or less (worst for the hippocampus and best for the white matter; similar across groups but slightly worse for the preterm group). Similar to the above results for the NeoBrainS12 dataset, given that the WUNDeR dataset had a voxel size of 1 mm isotropic, the current mean surface distances suggest that the MANTiS and manually edited segmentation boundaries are usually within one voxel of each other. Hausdorff distances were all around 14 mm or less (worst for the CSF and best for the brainstem; similar across groups but slightly worse for the preterm images).

**Table 3:** **Dice scores, mean surface distances (MSD) and Hausdorff distances (HD) for MANTiS compared with manually edited segmentations for the WUNDeR dataset**.

**Brain region/ Tissue type**	**Preterm (scanned at 27–32 weeks)**			**Preterm (scanned at TEA)**			**Term**			**All infants**		
	**Dice, mean (SD, sensitivity, specificity)**	**MSD, mean**	**HD, mean**	**Dice, mean (SD, sensitivity, specificity)**	**MSD, mean**	**HD, mean**	**Dice, mean (SD, sensitivity, specificity)**	**MSD, mean**	**HD, mean**	**Dice, mean (SD, sensitivity, specificity)**	**MSD, mean**	**HD, mean**
Cortical gray matter	0.81 (0.05, 0.97, 0.70)	0.43	8.19	0.86 (0.03, 0.96, 0.79)	0.29	7.64	0.89 (0.01, 0.92, 0.87)	0.18	12.41	0.86 (0.5, 0.95, 0.79)	0.30	9.41
White matter	0.92 (0.02, 0.86, 0.99)	0.37	7.05	0.91 (0.02, 0.88, 0.96)	0.24	8.98	0.92 (0.02, 0.91, 0.93)	0.19	8.44	0.92 (0.02, 0.88, 0.96)	0.27	8.16
Deep nuclear gray matter	0.89 (0.02, 0.95, 0.82)	0.71	11.61	0.88 (0.03, 0.95, 0.86)	0.63	6.83	0.91 (0.01, 0.94, 0.88)	0.64	6.23	0.90 (0.02, 0.95, 0.85)	0.66	8.22
Cerebellum	0.86 (0.03, 0.94, 0.79)	0.87	12.98	0.90 (0.03, 0.97, 0.83)	0.73	8.09	0.90 (0.01, 0.95, 0.88)	0.76	6.62	0.89 (0.03, 0.95, 0.83)	0.79	9.23
CSF	0.86 (0.04, 0.79, 0.95)	0.33	7.76	0.86 (0.03, 0.77, 0.97)	0.37	8.70	0.76 (0.04, 0.70, 0.85)	0.50	14.25	0.83 (0.06, 0.75, 0.92)	0.40	10.23
Brainstem	0.86 (0.04, 0.91, 0.81)	0.52	4.88	0.86 (0.02, 0.87, 0.84)	0.57	8.36	0.87 (0.01, 0.88, 0.87)	0.50	6.29	0.86 (0.03, 0.89, 0.84)	0.53	6.51
Hippocampus	0.69 (0.04, 0.42, 0.76)	1.32	11.16	0.66 (0.06, 0.55, 0.68)	0.98	8.40	0.67 (0.03, 0.58, 0.71)	0.82	7.45	0.67 (0.05, 0.52, 0.72)	1.04	9.00
Amygdala	0.54 (0.08, 0.66, 0.62)	0.94	9.64	0.51 (0.04, 0.74, 0.56)	0.92	10.75	0.53 (0.04, 0.66, 0.69)	0.82	4.76	0.53 (0.06, 0.69, 0.62)	0.89	8.38
Average	0.80 (0.04, 0.81, 0.81)	0.69	9.16	0.81 (0.03, 0.84, 0.81)	0.59	8.47	0.81 (0.02, 0.82, 0.84)	0.55	8.31			

*CSF, cerebrospinal fluid; TEA, term-equivalent age*.

Sensitivity of the Dice scores was calculated (Table 3). This measure corresponds to the true positive rate, i.e., the proportion of voxels that are correctly identified as the tissue type specified in the manually edited segmentations. Specificity of the Dice scores was also calculated (Table 3). This measure corresponds to the true negative rate, i.e., the proportion of voxels that are correctly identified as not being the tissue type specified in the manually edited segmentations. MANTiS generally had good sensitivity and specificity, as the average values were above 0.8 for all groups of infants and above 0.75 for all tissue types except for the hippocampus and amygdala, which had slightly lower sensitivity and specificity (Table 3).

Confusion matrices are presented in Tables 4–6 (one table for each group of infants), which show the overlap between the MANTiS segmentations and manually edited segmentations in terms of the percentage of voxels labeled as each brain region/tissue type. These confusion matrices show that the majority of voxels in the MANTiS segmentations were correctly labeled according to the manually edited segmentations.

**Table 4:** **Confusion matrix for the preterm (scanned at 27–32 weeks) images in the WUNDeR dataset**.

	**Brain region/Tissue type**	**Manually edited segmentations**								
		**CSF**	**Cortical GM**	**WM**	**DGM**	**Hippocampus**	**Amygdala**	**Cerebellum**	**Brainstem**	**Background**
MANTiS segmentations	CSF	95.23	0.90	0.82	0.28	0.15	0.01	0.50	0.29	1.83
	Cortical GM	11.17	70.03	15.13	0.19	0.54	0.23	0.02	0.00	2.67
	WM	0.97	0.20	98.71	0.06	0.05	0.01	0.00	0.00	0.00
	DGM	0.81	0.62	10.45	81.92	5.59	0.55	0.00	0.03	0.03
	Hippocampus	4.77	12.32	4.68	0.83	75.81	0.85	0.00	0.60	0.13
	Amygdala	0.64	4.96	8.80	2.32	21.00	62.22	0.00	0.00	0.05
	Cerebellum	14.62	1.80	0.10	0.00	0.00	0.00	79.51	0.72	3.25
	Brainstem	2.55	0.34	0.04	7.43	0.31	0.00	6.95	81.06	1.33

*CSF, cerebrospinal fluid; cortical GM, cortical gray matter; WM, white matter; DGM, deep nuclear gray matter. This table shows the overlap between MANTiS segmentations and manually edited segmentations in terms of percentage of voxels. For example, the first cell shows that 95.23% of voxels labeled CSF in the MANTiS segmentations were also labeled CSF in the manually edited segmentations, while the second cell (first row) shows that 0.90% of voxels labeled CSF in the MANTiS segmentations were labeled cortical GM in the manually edited segmentations*.

**Table 5:** **Confusion matrix for the preterm (scanned at term-equivalent age) images in the WUNDeR dataset**.

	**Brain region/Tissue type**	**Manually edited segmentations**								
		**CSF**	**Cortical GM**	**WM**	**DGM**	**Hippocampus**	**Amygdala**	**Cerebellum**	**Brainstem**	**Background**
MANTiS segmentations	CSF	97.01	1.21	0.39	0.15	0.05	0.00	0.31	0.23	0.65
	Cortical GM	9.52	78.89	10.33	0.10	0.25	0.11	0.02	0.00	0.77
	WM	1.91	1.97	95.89	0.12	0.07	0.01	0.01	0.00	0.00
	DGM	1.09	0.70	9.06	86.30	2.46	0.15	0.00	0.18	0.06
	Hippocampus	7.25	16.43	4.14	2.15	68.42	1.05	0.00	0.55	0.01
	Amygdala	2.26	4.42	16.63	8.39	12.62	55.48	0.00	0.00	0.20
	Cerebellum	11.24	3.08	0.14	0.01	0.00	0.00	83.53	1.06	0.94
	Brainstem	4.58	0.05	0.01	6.15	0.17	0.00	3.52	84.47	1.06

*CSF, cerebrospinal fluid; cortical GM, cortical gray matter; WM, white matter; DGM, deep nuclear gray matter. This table shows the overlap between MANTiS segmentations and manually edited segmentations in terms of percentage of voxels. For example, the first cell shows that 97.01% of voxels labeled CSF in the MANTiS segmentations were also labeled CSF in the manually edited segmentations, while the second cell (first row) shows that 1.21% of voxels labeled CSF in the MANTiS segmentations were labeled cortical GM in the manually edited segmentations*.

**Table 6:** **Confusion matrix for the term images in the WUNDeR dataset**.

	**Brain region/Tissue type**	**Manually edited segmentations**								
		**CSF**	**Cortical GM**	**WM**	**DGM**	**Hippocampus**	**Amygdala**	**Cerebellum**	**Brainstem**	**Background**
MANTiS segmentations	CSF	85.86	5.14	3.25	0.68	0.17	0.03	1.17	0.47	3.24
	Cortical GM	6.31	86.96	5.36	0.13	0.31	0.17	0.02	0.00	0.73
	WM	2.37	4.45	92.88	0.20	0.05	0.02	0.00	0.00	0.02
	DGM	0.56	1.55	8.56	87.56	1.29	0.39	0.00	0.08	0.01
	Hippocampus	5.98	13.63	3.82	3.01	70.52	2.70	0.00	0.31	0.04
	Amygdala	1.23	2.83	11.91	7.98	7.03	68.95	0.00	0.01	0.07
	Cerebellum	5.57	4.02	0.24	0.00	0.00	0.00	87.72	0.93	1.51
	Brainstem	2.87	0.06	0.00	5.94	0.06	0.00	3.39	87.17	0.51

*CSF, cerebrospinal fluid; cortical GM, cortical gray matter; WM, white matter; DGM, deep nuclear gray matter. This table shows the overlap between MANTiS segmentations and manually edited segmentations in terms of percentage of voxels. For example, the first cell shows that 85.86% of voxels labeled CSF in the MANTiS segmentations were also labeled CSF in the manually edited segmentations, while the second cell (first row) shows that 5.14% of voxels labeled CSF in the MANTiS segmentations were labeled cortical GM in the manually edited segmentations*.

### Errors in MANTiS classification

Misclassifications produced by MANTiS include the overestimation of the cortical gray matter in 30-week coronal scans (the worst scoring Dice score in the NeoBrainS12 dataset). This is a result of a combination of a number of effects, including anisotropic data and the template being very different to the novel data. The performance of MANTiS is lower for the preterm than the term-equivalent scans in both datasets (but particularly in the NeoBrainS12 dataset), which may be a reflection of the fact that the 40-week neonatal template is quite different in appearance to the preterm brain images. MANTiS may perform better for such cases if a more representative template is used, such as one derived from 30-week scans. However, the Dice scores for preterm scans in the WUNDeR dataset were still good (similar to the term-equivalent scans), which suggests that the anisotropic coronal acquisition for the NeoBrainS12 scans may have had a strong effect on the results. MANTiS' worse performance for the preterm and coronal acquisitions is highlighted in Figure 6, where the distribution of Dice scores is wider for the preterm scans compared with the term scans, and slightly wider for the coronal term scans compared with the axial term scans. Overestimation of cortical gray matter is more evident in preterm than term scans, however MANTiS may also slightly overestimate cortical gray matter even in term scans, especially in complexly folded cortical regions where there is extensive fusing of sulcal CSF that may be labeled as gray matter; this issue may be related to limited image resolution and the fact that CSF is quite thin in these regions. This overestimation of sulci at the expense of extracerebral CSF was also found to occur in other methods that participated in the NeoBrainS12 challenge, highlighting the difficulty in segmenting the sulci and the need for further work to refine sulci segmentation (Isgum et al., 2015). Other errors made by MANTiS include mislabeling of darker (myelinated) white matter as either cortical or deep nuclear gray matter, such as in the posterior limb of the internal capsule and parts of the corpus callosum, and classification of scalp voxels as cortical gray matter, which could be alleviated by inputting images with a better quality brain extraction.

There were three common types of misclassification of cortical gray matter in the WUNDeR images that contributed to the Hausdorff distance measures. In 30-week scans, hypo-intense white matter was sometimes misclassified as small, isolated gray matter regions. In 40-week scans, dura that was not removed during brain extraction, and voxels on the edges of the hippocampus and amygdala, were sometimes misclassified as cortical gray matter. The most common type of misclassification of white matter that contributed to the Hausdorff distance measures was misclassification of voxels on the ventricle boundaries and along the midline in the vicinity of the choroid plexus and extra-ventricular CSF as white matter. Hausdorff distances for brainstem were driven by misclassification of isolated peripheral voxels as brainstem (false positives), while Hausdorff distances for cerebellum were driven by a combination of misclassified peripheral voxels and misclassification of hyper-intense cerebellar voxels as CSF.

## Discussion

This paper has described and validated MANTiS, a novel method for tissue classification of brain MR images of neonates. MANTiS uses the established unified segmentation method for adult brain tissue classification, a neonatal atlas and morphological segmentation and filtering to segment neonatal MR images into eight tissue types or brain regions; the cortical gray matter, white matter, deep nuclear gray matter, CSF, brainstem, cerebellum, hippocampus, and amygdala.

The evaluation results using the NeoBrainS12 and WUNDeR datasets show that MANTiS produces segmentations with good agreement with manual and manually edited segmentations, with similar Dice scores across datasets, despite differences in acquisition and manual segmentation protocols. The evaluation using the NeoBrainS12 dataset enabled us to perform a comparison between our method and the other methods that took part in the NeoBrainS12 challenge. Our Dice scores, as well as mean surface distances and Hausdorff distances, for comparison between the MANTiS segmentations and manual segmentations of preterm MR images were competitive with the scores obtained by the other methods that participated in the challenge (Isgum et al., 2015). Hausdorff distances were driven by isolated false positive voxels for most classes, having little impact on volume estimates. The evaluation results also showed that MANTiS performed well for infants at variable maturational stages; preterm infants scanned shortly after birth, preterm infants scanned at term-equivalent age, and importantly, term-born infants, which were not assessed as part of the NeoBrainS12 challenge. The pipeline involved identical processing and tissue priors for all infants—i.e., identical for NeoBrainS12 and WUNDeR data, and agreement with manual or manually edited segmentations was good for all groups of infants. This demonstrates the remarkable adaptability of MANTiS given the many changes that occur between 27 weeks' gestational age and term-equivalent age, and the many differences between preterm and term-born infants at term-equivalent age, in terms of brain size, structure, and shape (Huppi et al., 1998; Tao and Neil, 2014; Anderson et al., 2015).

MANTiS also performed well visually for MR images with various brain abnormalities, including enlarged, distorted ventricles and white matter signal intensity abnormalities, without user intervention. An example of a MANTiS segmentation in a preterm infant with severe brain abnormalities is shown in Figures 5A,B; this infant had bilateral IVH grade 3 and cystic PVL, and on the term-equivalent scan enlarged, distorted ventricles, enlarged interhemispheric distance and reduced white matter volume can be seen. The images included in the validation datasets had no brain abnormalities or mild-moderate brain abnormalities; therefore it may be beneficial to further validate MANTiS using images with more severe brain abnormalities.

MANTiS has modest technical requirements and can be run on computers at any institution. Processing time (approximately 7 min without brain extraction; approximately 15 min including brain extraction using FSL's BET) is similar to or less than that reported for other automated neonatal brain tissue classification methods. Of the teams that participated in the NeoBrainS12 challenge, 1 team's method required 7 min, 3 teams' methods required 15 min, and the remaining 4 teams' methods required an hour or more (Isgum et al., 2015). However processing time is difficult to compare across studies as it is depends on the computer used. MANTiS also performs well for images acquired from different scanners and hospitals, as evidenced by our validation results using the two datasets acquired from different scanners and hospitals.

### Limitations

We acknowledge that currently available brain masking methods, such as FSL's BET, which was used in the current study, have been designed for adult MR images and may not always be accurate for neonatal MR images. Development of brain masking methods for neonatal images may improve future studies, and a preliminary version of an alternative brain masking method for neonatal images is available in the MANTiS toolbox. For the evaluation of our results using the images in the WUNDeR dataset, we created a reference standard by automatically segmenting MR images and then manually editing these automatic segmentations. The initial segmentation by an automatic method may introduce some bias into the reference standard, which may affect the Dice scores, however it enabled us to create a reference standard in a large number of images (*n* = 36 individual images). However, the use of multiple raters is likely to increase variability in manually edited segmentations compared with a single rater, possibly reducing the maximum Dice scores. For the evaluation of our results using the NeoBrainS12 dataset, a purely manual reference standard was created. Our Dice scores were similar for the WUNDeR and NeoBrainS12 datasets, which suggests that pre-segmentation by the automatic method did not affect our validation results for the WUNDeR dataset. Some of the other NeoBrainS12 methods were able to segment separately the myelinated and unmyelinated white matter, while MANTiS combines the myelinated and unmyelinated white matter into one tissue type. However, it was concluded from the NeoBrainS12 challenge that the myelinated white matter was the least accurately segmented brain tissue class (Isgum et al., 2015). Accurate white matter segmentation, as generated by MANTiS, may be a useful first step in carrying out the separate segmentation of myelinated and unmyelinated white matter. Additionally, MANTiS does not segment the CSF in the ventricles and extracerebral space separately, which may be beneficial in future work. MANTiS is able to segment the hippocampus and amygdala, however the Dice scores for these structures were poorer than for the other brain regions/tissue types, likely due to their small size. Therefore, the hippocampus and amygdala segmentations produced by MANTiS should be interpreted with caution and possibly combined with the deep nuclear gray matter or cortical gray matter segmentations.

## Conclusion

We have introduced MANTiS, a new, fast, fully automated method for neonatal brain tissue classification that utilizes a combination of a well-established method for adult brain tissue segmentation, a recent neonatal atlas, and novel morphological segmentation methods. The combination of the well known unified segmentation method with data-driven adaptation via watershed segmentation and topological filtering provides both stability and adaptability and eliminates separate registration steps. Tissue classification provides a basis for many subsequent analyses, such as volumetric and shape analyses, cortical parcellation and cortical thickness measurement, and therefore MANTiS will contribute significantly to studying typical and atypical neonatal brain development. MANTiS is available for download at http://developmentalimagingmcri.github.io/mantis.

## Author contributions

Conceived and designed the study: RB, JC, JLYC, AS, PA, LD, TI, MS, DT; Acquired, analyzed or interpreted data for the study: RB, JC, CK, DA, CS, CR, WL, LM, JLYC, AS, PA, LD, TI, MS, DT; Drafted or critically revised the paper and approved the final version: RB, JC, CK, DA, CS, CR, WL, LM, JLYC, AS, PA, LD, TI, MS, DT.

## Funding

This work was supported in part by the Australian National Health and Medical Research Council (NHMRC) (Project Grant ID 1028822; Centre of Clinical Research Excellence Grant ID 546519; Centre of Research Excellence Grant ID 1060733; Senior Research Fellowship ID 1081288 to PA; Early Career Fellowship ID 1053787 to JLYC, ID 1053767 to AS, ID 1012236 to DT), Murdoch Childrens Research Institute, Clinical Sciences Theme Grant, the Victorian Government Operational Infrastructure Support Program, The Royal Children's Hospital Foundation, National Institutes of Health (NIH) grants R01 HD05709801 (TI), UL1 TR000448 (CS and CR), K02 NS089852 (CS), and K23 MH105179 (CR), and the Mallinckrodt Institute of Radiology (CS). The funding sources had no involvement in study design; in the collection, analysis and interpretation of data; in the writing of the report; and in the decision to submit the article for publication.

### Conflict of interest statement

The authors declare that the research was conducted in the absence of any commercial or financial relationships that could be construed as a potential conflict of interest.
